# Associations of sedentary behavior and physical activity with sleep disorders in patients with osteoarthritis: The mediating role of inflammatory biomarkers

**DOI:** 10.1016/j.ocarto.2026.100756

**Published:** 2026-02-08

**Authors:** Bin Luo, RuiZe Chen, Zongyuan Huang, Shuai Zhang, Yanting Chen, Mingde Cao, Ling Kong, Xin Wu, Qianshi Guo, Yuxiang Ouyang, Hua Wang, Yancheng Song

**Affiliations:** a Department of Orthopedics, The First Affiliated Hospital of Guangdong Pharmaceutical University, Guangdong Pharmaceutical University, No. 19 Nonglinxia Road, Yuexiu District, Guangzhou, Guangdong Province, China; b Guangzhou Regenerative Medicine and Health Guangdong Laboratory, Guangzhou, China; c Department of Orthopaedics and Traumatology, Faculty of Medicine, The Chinese University of Hong Kong, Shatin, Hong Kong

**Keywords:** Osteoarthritis, Sleep disorders, Inflammation, Physical activity, Sedentary behavior, C-reactive protein

## Abstract

**Objective:**

This study examines how physical activity and sedentary behavior influence sleep disturbances in OA patients, with inflammation potentially mediating these effects.

**Methods:**

Data related to OA, sleep disorders, sleep quality, inflammatory markers, physical activity and sedentary behavior from 4386 adults were collected from the NHANES. Mediation analysis evaluated inflammation's role.

**Results:**

Sedentary behavior increased sleep disorder risk in OA patients, while physical activity was protective. Inflammatory markers (notably CRP and NLR) mediated 5.3 %–17.9 % of the association between OA and sleep disorders. Physical activity's protective effect was stronger in highly sedentary individuals.

**Conclusion:**

Reducing sedentary behavior and increasing physical activity may improve sleep in OA, potentially via inflammatory pathways. Further longitudinal research is warranted.

## 1 Introduction

Sleep disorders refer to conditions that affect sleep patterns and have a negative impact on overall health, including types such as insomnia, sleep apnea, restless legs syndrome, and narcolepsy [1,2]. According to a survey by the World Health Organization, approximately 27 % of the global population suffers from sleep problems, and sleep disorders have become an increasingly serious public health issue worldwide. In related studies conducted in the United States, osteoarthritis was found often co-occurring with sleep disorders [3].

Osteoarthritis (OA) is one of the leading causes of disability worldwide, with an estimated 540 million people affected globally [4]. This chronic joint disease not only leads to degenerative damage to the articular cartilage and surrounding tissues but also causes pain, stiffness, and loss of joint function, significantly impacting patients' quality of life [5]. In China, the number of OA cases reached 10,681,311 in 2019, an increase of 132.66 % compared to 1990. Additionally, the disability-adjusted life years (DALY) caused by OA reached 4,724,885, indicating its significant impact on the societal health burden [6,7]. Given the growing prevalence and profound impact of OA, it is crucial to prioritize research on OA patients.

Sedentary behavior, common in OA patients due to joint pain or movement avoidance, amplifies inflammation by reducing muscle metabolism, accumulating adipose tissue, and impairing pro-inflammatory mediator clearance, thereby elevating markers like CRP and NLR; this inflammation further disrupts sleep, possibly via HPA axis dysregulation (suppressing melatonin) or heightened pain, forming an inactivity-inflammation-poor sleep cycle. In contrast, OA-tailored physical activity (e.g., brisk walking, swimming) acts protectively: it suppresses pro-inflammatory cytokines (e.g., TNF-α), releases anti-inflammatory myokines, and reduces adipose tissue to ease inflammation's impact on sleep, with greater benefits in highly sedentary OA patients (even small activity increases may help).

Inflammation is considered a trigger for sleep disorders [8]. Anti-inflammatory treatments, including non-steroidal anti-inflammatory drugs, cytokine inhibitors, statins, corticosteroids, and minocycline (a microglial cell inhibitor), have been found to significantly improve the effectiveness of treatments for both sleep disorders [9,10]. Although the role of inflammation in the comorbidity of sleep disorders has received attention, many unresolved mysteries remain. In this study, we utilized various inflammatory markers, including the neutrophil-to-lymphocyte ratio (NLR), an economically effective marker that reflects inflammation levels through a complete blood count; additionally, we used C-reactive protein (CRP), a common acute-phase plasma protein, to further assess the extent of the inflammatory response [11,12]. While existing studies have established the relationship between physical activity, sedentary behavior, and sleep disorders [13], the role of modifiable lifestyle factors—physical activity and sedentary behavior—in this inflammatory-driven relationship remains understudied in large OA-focused population research.

Therefore, this cross-sectional study aims to: i) use National Health and Nutrition Examination Survey samples to explore the association between physical activity and sedentary behavior and sleep disorders; ii) elucidate how blood cell-based inflammatory biomarkers influence sleep disorders in OA patients; iii) investigate whether these inflammatory biomarkers mediate the relationships between physical activity/sedentary behavior and sleep disorders.

## 2 Materials and methods

### 2.1 Data sources

**Fig. 1:**
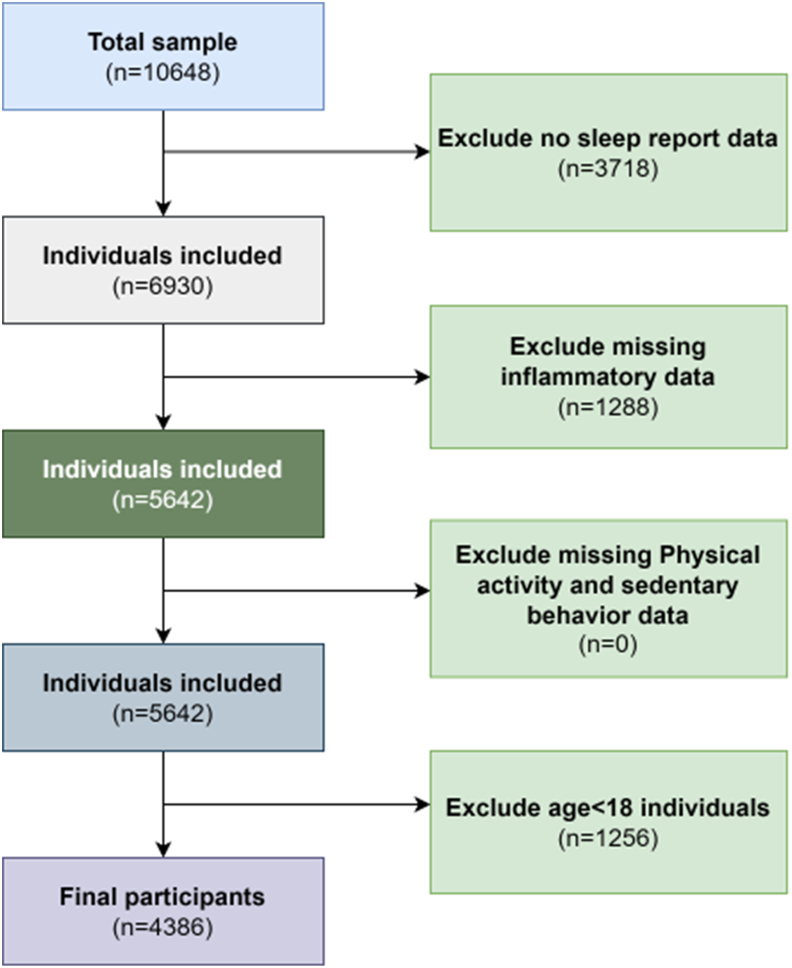
Flowchart of the study design and participants excluded from the study.

This study used data from the National Health and Nutrition Examination Survey (NHANES). NHANES employs a complex, multi-stage probability sampling design to ensure that the selected sample is representative of the U.S. general population. The survey assesses the health and nutritional status of individuals across various age groups through interviews and medical examinations. The NHANES interview collects information on participants' general health, socioeconomic status, and demographic characteristics, while the medical examination includes physical assessments (such as anthropometry), clinical measurements, and biological specimen collection (such as blood and urine) for laboratory analysis. All examinations are conducted in specially equipped, fully outfitted mobile examination centers (MECs). NHANES data are publicly released every two years. We selected the 2015–2023 data because adjustments were made for oversampled subgroups during this period to better capture inflammatory markers associated with osteoarthritis, enabling the investigation of their relationship with sleep disorders. The participants in this study were adults (≥18 years old) who participated in the NHANES survey from 2015 to 2023. Participants included in this study were adults (≥18 years old) who participated in the NHANES survey from 2015 to 2023, were not pregnant, diagnosed with osteoarthritis, and provided complete data on sleep duration, sleep disorders, inflammatory markers, and covariates. After applying the eligibility criteria mentioned above, we ultimately obtained 4386 analytical samples (Fig. 1).

### 2.2 Evaluation of osteoarthritis

Information regarding osteoarthritis was obtained from participants' self-reported responses to the following questions: "Have you ever been diagnosed with osteoarthritis by a doctor?" and "In the past 12 months, have you sought medical help for joint pain or stiffness?" If participants answered "yes" or provided relevant medical diagnosis records, they were classified as having osteoarthritis; if they answered "no" or did not provide relevant records, they were considered not to have osteoarthritis [14,15]. This classification method provides a reliable basis for analyzing the relationship between osteoarthritis and health outcomes.

### 2.3 Biomarkers

The NLR is obtained by calculating the ratio of neutrophil count to lymphocyte count. Neutrophil and lymphocyte counts are measured using the Beckman Coulter MAXM instrument (10ˆ9 cells/L). CRP was quantified using the latex-enhanced turbidimetry method on the Behring instrument, with a lower limit of detection (LLOD) of 0.02 mg/dL. During the 2015–2023 NHANES period, CRP levels were measured using the Beckman UniCel analyzer, with lower limits of 0.011 mg/dL (2015–2016) and 0.015 mg/dL (2017–2018); values below the LLOD were replaced with LLOD divided by the square root of 2 (LLOD/sqrt) [16].

The primary mediating variables are inflammatory biomarkers obtained from blood samples of participants collected from the NHANES database. We used inflammatory biomarkers associated with osteoarthritis and sleep disorders, including C-reactive protein (CRP), lymphocytes (LYM), neutrophils (NEU), and white blood cell count (WBC). The method for collecting inflammatory biomarkers in the NHANES data remained consistent throughout the study period, and the data collection process was thoroughly ensured. For example, the collection of CRP, lymphocytes, neutrophils, and WBC was completed thoroughly from 2015 to 2023, with relevant medical permissions obtained. Based on standardized cut-off values from previous studies, the data for inflammatory biomarkers were classified as abnormal or normal. The specific cut-off values are: CRP ≥3 mg/L, WBC ≥10 × 10ˆ9/L, LYM ≥4.0 × 10ˆ9/L, NEU ≥7.0 × 10ˆ9/[17]. Participants with abnormal levels were classified as having elevated inflammatory biomarkers. This classification method provides a standardized basis for analyzing the potential association between osteoarthritis and sleep disorders and clarifies the mediating role of inflammatory biomarkers in this relationship.

### 2.4 Sleep disorders

Information regarding sleep disorders was obtained from participants' self-reported responses to the following question: "In the past month, how often have you been bothered by the following issues: difficulty falling asleep, difficulty staying asleep, waking up frequently during the night, or waking up too early and not being able to fall back asleep?" If participants answered "more than half of the time" or "almost every day," they were classified as having sleep disorders; if they answered "occasionally" or "never," they were considered not to have sleep problems. Additionally, to further confirm the presence of sleep disorders, we included participants' reported sleep duration (<6 h) and subjective sleep quality ratings (poor or very poor) as supplementary indicators [18,19].

### 2.5 Physical activity and sedentary behavior

As primary exposure variables, data on physical activity and sedentary behavior were obtained from self-reported responses to the NHANES questionnaire. Since 2015, NHANES has updated questions related to physical activity, using the standards for moderate and vigorous-intensity recreational activities to assess activity levels, measured in minutes per week. Relevant questions include: "During a typical week, have you engaged in any vigorous exercise, fitness, or recreational activities, such as running or playing basketball, that significantly increased your breathing rate or heart rate and lasted for more than 10 min?" and "During a typical week, have you engaged in any moderate-intensity activities, such as brisk walking, cycling, swimming, or volleyball, that mildly increased your breathing rate or heart rate and lasted for at least 10 min?" [20].

Regarding sedentary behavior, the assessment was based on the question, "How much time do you typically spend sitting each day?" Sedentary behavior was defined as the time spent sitting still in places such as school, home, or during commuting, such as working at a desk, using transportation, reading, playing cards, watching television, or using electronic devices, but excluding sleep time [20]. Based on participants' self-reports, the classification criteria for sedentary behavior are as follows: individuals with daily sedentary time exceeding 480 min are defined as having high sedentary behavior. In comparison, those with less than 480 min are classified as having low sedentary behavior.

According to the World Health Organization's recommendations on physical activity, performing more than 150 min of moderate-intensity aerobic exercise per week, or more than 75 min of vigorous-intensity aerobic exercise, or an equivalent combination of moderate and vigorous-intensity activities (with 1 min of vigorous-intensity exercise equivalent to 2 min of moderate-intensity exercise), totaling 150 min or more, is defined as moderate/high physical activity. Otherwise, it is classified as low physical activity [21].

### 2.6 Covariates

We selected the relevant covariates discussed in previous studies exploring the relationship between osteoarthritis and sleep disorders, and considered demographic characteristics known to be associated with inflammatory biomarkers and sleep disorders. The sociodemographic variables associated with inflammatory biomarkers and sleep disorders include age (18–44 years, 44–60 years, 60 years and above), gender (male, female), race/ethnicity (Hispanic, non-Hispanic White, non-Hispanic Black, other races), education level (high school or below, some college education, associate degree or higher), marital status (single, married, divorced/widowed/separated, living with a partner), BMI (<25, [25,30), ≥30), alcohol use (yes/no), smoking habits (yes/no), physical activity level (sedentary/moderate intensity/vigorous intensity), and the presence of comorbidities (yes/no). Alcohol use status was collected through two 24-h dietary recall questionnaires. If participants reported alcohol consumption in at least one of the 24-h recalls, they were classified as alcohol users. Smoking status was assessed using the following categories: never smoked (smoked <100 cigarettes), former smoker (smoked in the past but not currently, and smoked ≥100 cigarettes), current smoker (currently smoking and smoked ≥100 cigarettes, and smoking daily or on some days). Poverty was assessed using the poverty income ratio, where a PIR of ≤1 indicates households below the poverty line, 1–3 represents moderate-income households, and >3 refers to households with income exceeding three times the poverty line. If participants reported any of the following health conditions: diabetes, renal failure, kidney stones, heart failure, stroke, liver disease, rheumatoid arthritis, or cancer, they were classified as having comorbidities. For missing data, a random forest-based imputation method was used. These factors were controlled for as potential confounding variables during the analysis.

### 2.7 Statistical analysis

Statistical analysis was performed using R software (version 4.2.0; https://cran.r-project.org/) and relevant packages (such as interaction and mediation analysis). In all analyses, a two-tailed p-value <0.05 was considered statistically significant. In descriptive statistics, continuous variables are presented as mean and standard deviation or median and interquartile range, while categorical variables are expressed as frequency and percentage. The comparison of categorical variables between groups was performed using the χ^2^ test. For continuous variables, data that followed a normal distribution were compared between groups using one-way analysis of variance (ANOVA), while data with skewed distributions were compared using the Kruskal-Wallis H test.

Baseline characteristics between the sleep disturbance and non-disturbance groups were compared using independent sample t-tests for continuous variables and chi-square tests for categorical variables. To explore the association between sleep disturbance, physical activity, sedentary behavior and inflammatory biomarkers, multivariate logistic regression was performed. Odds ratios (OR) and 95 % confidence intervals (CI) were calculated. The unadjusted model did not control for any confounders; in model 1, adjustments were made for age, sex, and race/ethnicity; and in model 2, additional adjustments were made for age, sex, race, body mass index (BMI), marital status, sleep duration, education level, smoking, and alcohol consumption. Poverty income ratio was calculated as the ratio of family income to the poverty threshold specific to family size, year, and state.

Mediation analysis was performed using the mediation package in R software, which quantifies the mediation effect and provides insights into potential pathways, supporting the understanding of underlying mechanisms. In this study, the direct effect represented the relationship between sedentary behavior, physical activity, and sleep disturbance, while the indirect effect was the relationship mediated by inflammatory markers. The proportion mediated indicates the percentage of the total effect that is accounted for by the mediation. All analyses were conducted using R (version 4.2.0, http://www.R-project.org, The R Foundation), with statistical significance defined as P < 0.05.

## 3 Result

This study ultimately included 4386 participants, with a weighted total of 4386 participants. The weighted prevalence of sleep disorder symptoms was 23.71 %. The average age of the participants was 41.39 ± 15.15 years, and 56.06 % of the participants were male (Table 1). Specifically, the weighted prevalence of sleep disorders was higher in the male group, accounting for 55.19 % of all participants. Furthermore, after stratified analysis by age, gender, race, body mass index, marital status, sleep duration, education level, smoking status, and alcohol use, the differences in the weighted prevalence of sleep disorders were statistically significant (P < 0.05). These results suggest that the prevalence of sleep disorders varies significantly across different population characteristics, indicating that the condition may be influenced by multiple factors.

**Table 1:** Weighted characteristics of the study population by sleep disturbance status.

Variable	N	Overall	no	yes	p-value
		N = 4386	N = 3346	N = 1040	
LLOD, mean ± sd	4386	2.17 ± 4.11	2.07 ± 4.02	2.50 ± 4.35	0.004
NLR, mean ± sd	4386	2.00 ± 1.11	1.99 ± 1.13	2.01 ± 1.04	0.611
NEU, mean ± sd	4386	3.97 ± 1.62	3.93 ± 1.61	4.07 ± 1.66	0.018
Physical activity, n (p%)	4386				0.046
Mild		2957.00 (67.42 %)	2229.00 (66.62 %)	728.00 (70.00 %)	
Severe		1429.00 (32.58 %)	1117.00 (33.38 %)	312.00 (30.00 %)	
Sedentary_behavior, n (p%)	4386				<0.001
Light		3503.00 (79.87 %)	2722.00 (81.35 %)	781.00 (75.10 %)	
Vigorous/moderate		883.00 (20.13 %)	624.00 (18.65 %)	259.00 (24.90 %)	
BMI, n (p%)	4386				<0.001
[25.30)		1555.00 (35.45 %)	1202.00 (35.92 %)	353.00 (33.94 %)	
<25		1394.00 (31.78 %)	1155.00 (34.52 %)	239.00 (22.98 %)	
≥30		1437.00 (32.76 %)	989.00 (29.56 %)	448.00 (43.08 %)	
Smokers, n (p%)	4386				<0.001
Don not		2.00 (0.05 %)	2.00 (0.06 %)	0.00 (0.00 %)	
No		2956.00 (67.40 %)	2377.00 (71.04 %)	579.00 (55.67 %)	
Yes		1428.00 (32.56 %)	967.00 (28.90 %)	461.00 (44.33 %)	
Sex, n (p%)	4386				0.540
Female		1927.00 (43.94 %)	1461.00 (43.66 %)	466.00 (44.81 %)	
Male		2459.00 (56.06 %)	1885.00 (56.34 %)	574.00 (55.19 %)	
Age, n (p%)	4386				<0.001
[44, 60)		1071.00 (24.42 %)	773.00 (23.10 %)	298.00 (28.65 %)	
<44		2654.00 (60.51 %)	2129.00 (63.63 %)	525.00 (50.48 %)	
≥60		661.00 (15.07 %)	444.00 (13.27 %)	217.00 (20.87 %)	
Race, n (p%)	4386				<0.001
Mexican American		626.00 (14.27 %)	484.00 (14.47 %)	142.00 (13.65 %)	
Non-Hispanic Black		1447.00 (32.99 %)	1041.00 (31.11 %)	406.00 (39.04 %)	
Non-Hispanic White		1001.00 (22.82 %)	776.00 (23.19 %)	225.00 (21.63 %)	
Other race/ethnicity		1312.00 (29.91 %)	1045.00 (31.23 %)	267.00 (25.67 %)	
Education, n (p%)	4386				0.031
Elow high school		1046.00 (23.85 %)	814.00 (24.33 %)	232.00 (22.31 %)	
High school		1481.00 (33.77 %)	1095.00 (32.73 %)	386.00 (37.12 %)	
Other race/ethnicity		1859.00 (42.38 %)	1437.00 (42.95 %)	422.00 (40.58 %)	
Marita, n (p%)	4386				0.004
Never married		460.00 (10.49 %)	364.00 (10.88 %)	96.00 (9.23 %)	
Married/living with partner		1007.00 (22.96 %)	778.00 (23.25 %)	229.00 (22.02 %)	
Widowed/divorced		196.00 (4.47 %)	130.00 (3.89 %)	66.00 (6.35 %)	
Don not		2723.00 (62.08 %)	2074.00 (61.98 %)	649.00 (62.40 %)	
Poverty, n (p%)	4386				0.077
[1, 3)		1278.00 (29.14 %)	1000.00 (29.89 %)	278.00 (26.73 %)	
<1		495.00 (11.29 %)	373.00 (11.15 %)	122.00 (11.73 %)	
≥3		2043.00 (46.58 %)	1527.00 (45.64 %)	516.00 (49.62 %)	
999		570.00 (13.00 %)	446.00 (13.33 %)	124.00 (11.92 %)	
Osteoarthritis, n (p%)	4386				<0.001
No		3686.00 (84.04 %)	2962.00 (88.52 %)	724.00 (69.62 %)	
Yes		700.00 (15.96 %)	384.00 (11.48 %)	316.00 (30.38 %)	

This study constructed three weighted logistic regression models to assess the relationship between sedentary behavior, physical activity, and sleep disorders in osteoarthritis patients (Table 2). In the preliminary model, the odds ratio (OR) for sleep disorders in osteoarthritis participants was 0.244, with a 95 % confidence interval (CI) of 0.225–0.265 (P < 0.001) (Model 1). After further adjusting for basic factors such as age, gender, and race (Model 2), the trend remained stable. With additional adjustments for body mass index, marital status, sleep duration, education level, poverty income ratio, smoking status, and physical activity and sedentary behavior (Model 3), the OR for the sedentary behavior group was 10.704, with a 95 % CI of 8.801–14.814 (P < 0.001), while the OR for the physical activity group was 8.674, with a 95 % CI of 5.744–12.864 (P < 0.001). These results suggest that there is a positive correlation between sedentary behavior and sleep disorders, while moderate physical activity may help reduce the risk of sleep disorders.

**Table 2:** Study on the mechanism of influence of long-term sitting and physical activity on sleep disorders in patients with osteoarthritis under the mediation of inflammatory markers.

	Crude modela		Model 1b		Model 2c		OR	95 % CI	P-value
	OR (95 % CI)	P-value	OR (95 % CI)	P-value	OR (95 % CI)	P-value			
Mild sedentary behavior	Reference		Reference		Reference			Reference	
Severe sedentary behavior	1.724 (1.220,2.419)	<0.001	5.019 (3.218,8.076)	<0.001	10.704 (8.801,14.814)	<0.001	2.654	(1.569,3.211)	<0.004
Light physical activity			Reference		Reference			Reference	
Vigorous/moderate physical activity	0.325 (0.273,0.387)	<0.001	4.473 (2.872,7.168)	<0.001	8.674 (5.744,12.864)	<0.001	2.455	(1.269,3.012)	<0.012
Osteoarthritis	0.244 (0.225,0.265)	<0.001	0.390 (0.307,0.493)	<0.001	0.410 (0.350,0.520)	0.009	0.687	(0.551,0.857)	0.002

OR, odds ratio; CI, confidence intervals.

Crude model: no covariates were adjusted.

Model 1: age, sex, and race/ethnicity were adjusted.

Model 2: age, sex, race, body mass index, marital status, sleep duration, education attainment, poverty income ratio, smoking status, and alcohol drinking status were adjusted.

In addition, we further explored the relationship between physical activity and sleep disorders in osteoarthritis patients under different levels of sedentary behavior. After adjusting for all covariates, the results showed that for the low sedentary behavior group, although there was a negative correlation between physical activity and sleep disorders, the association between physical activity intensity and sleep disorders was not significant. In contrast, in the high sedentary behavior group, higher physical activity intensity was significantly associated with a lower risk of sleep disorders (OR = 2.455, 95 % CI: 1.269–3.012, P < 0.012) (Table 2). This result suggests that for individuals with sedentary behavior, increasing physical activity intensity may have a protective effect, significantly reducing the risk of sleep disorders.

**Fig. 2:**
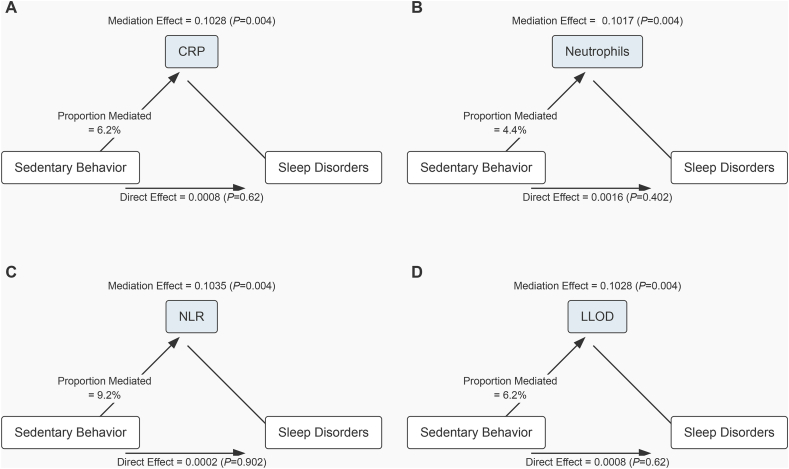
Path diagram of the mediation analysis of inflammatory biomarkers on the relationship between Sedentary behavior and sleep disorders. The graphs in (A–D) represented the mediating role of CRP,Neutrophils,NLR and LLOD.

**Fig. 3:**
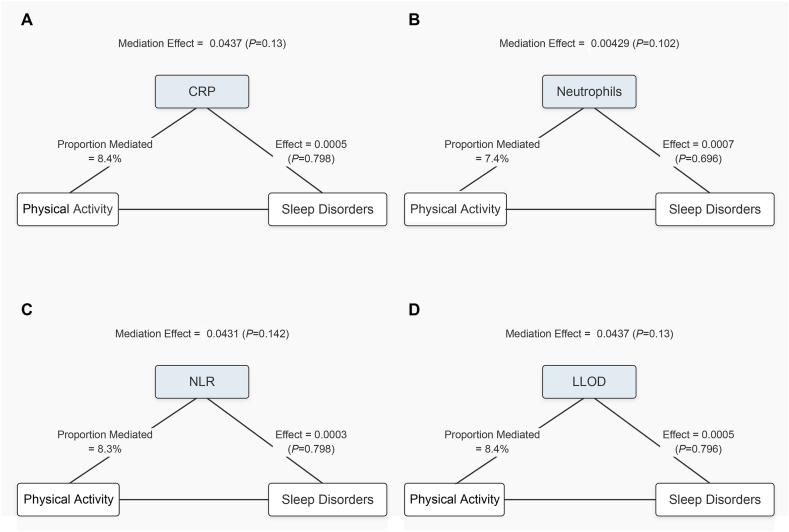
Path diagram of the mediation analysis of inflammatory biomarkers on the relationship between Physical activity and sleep disorders. The graphs in (A–D) represented the mediating role of CRP, Neutrophils, NLR and LLOD.

In further analysis, we explored the mediating role of inflammatory biomarkers in the relationship between sedentary behavior and sleep disorders (Fig. 2). The results show that all four inflammatory biomarkers (CRP, NEU, NLR, and LLOD) significantly mediated. Specifically, CRP, neutrophils, NLR, and LLOD explained 3.8 %, 2.3 %, 9.0 %, and 3.6 % of the mediation effect, respectively (P < 0.05). Furthermore, we conducted a mediation analysis (Fig. 3). The results showed that CRP, NEU, NLR, and LLOD explained 8.4 %, 7.4 %, 8.3 %, and 8.4 % of the mediation effect between physical activity and sleep disorders, respectively. Although the direct effects of these four inflammatory biomarkers were all significant (P < 0.001), in some cases, the mediating effects of inflammatory biomarkers did not show significant influence.

## 4 Discussion

The aim of this study is to investigate the association between sedentary behavior, physical activity, and sleep disorders in osteoarthritis patients, and to determine whether this association is partially mediated by inflammatory markers in the blood. First, our findings suggest that sedentary behavior and physical activity are related to sleep disorders. Specifically, prolonged sedentary behavior may be positively correlated with sleep disorders, while appropriate physical activity may be negatively correlated with sleep disorders. However, the relationships between these factors may vary in women, with stronger correlations observed.

Sleep disorders are common in osteoarthritis patients and can affect their quality of life and disease recovery process. Due to physiological factors such as changes in estrogen levels, women may be more susceptible to the effects of sedentary behavior and physical activity during osteoarthritis, and the likelihood of experiencing sleep disorders may be higher compared to men [[22], [23], [24]]. Furthermore, the results show that sleep disorders are related to higher levels of CRP, NEU, NLR, and LLOD, while improvements in sleep status due to moderate physical activity may be associated with lower inflammatory markers [25]. Previous studies have shown that osteoarthritis patients experience elevated levels of blood cell markers (such as CRP, NEU, NLR, and LLOD), leading to a chronic low-grade inflammatory state [26]. These inflammatory mediators can spread throughout the body via the bloodstream, affecting central nervous system function. Long-term static states of the body (such as sedentary behavior) and abnormal states like excessive fatigue can activate inflammatory signaling pathways, such as the mitogen-activated protein kinase pathway and the JAK-STAT pathway.

The following mechanisms can further explain the relationship between sedentary behavior, physical activity, inflammatory markers, and sleep disorders: First, prolonged sedentary behavior leads to reduced skeletal muscle metabolic activity, fat tissue accumulation, and promotes the secretion of adipokines (such as leptin and resistin), which further stimulate monocytes to release IL-6 and CRP, as well as increase WBC, NEU, NLR, and LLOD levels in a positive correlation. Studies have shown that sedentary time is positively correlated with serum CRP levels [27,28]. Sedentary behavior causes the body to remain in a low metabolic state for prolonged periods, with reduced joint activity, which may result in poor local blood circulation and the accumulation of metabolic products in the periarticular tissues, stimulating an inflammatory response in the body, thereby affecting sleep quality [29]. On the other hand, regular physical activity reduces inflammation through the following pathways: physical activity induces an acute increase in IL-6 (which has both pro-inflammatory and anti-inflammatory effects), long-term suppression of chronic inflammatory markers such as TNF-α, promotes the release of muscle factors, or reduces the secretion of pro-inflammatory adipokines, leading to a reduction in fat tissue [30]. Regular physical activity enhances the stability of the nervous system, regulates the endocrine system, suppresses inflammatory responses, and benefits sleep quality.

Previous studies have examined the relationship between lifestyle behaviors (sedentary behavior, physical activity), inflammatory markers, and sleep disorders, but findings have been inconsistent. Elevated CRP, NEU, NLR, and LLOD levels, which are associated with increased inflammation, can pass through the blood-brain barrier, suppressing the hypothalamic-pituitary-adrenal (HPA) axis, reducing melatonin secretion, and disrupting the sleep-wake cycle. These markers are independent risk factors for sleep disorders [31]. In OA patients, prolonged sedentary behavior exacerbates joint stiffness, pain, and muscle atrophy, while slowing blood circulation and impairing the clearance of inflammatory cytokines, leading to elevated inflammatory markers. These high levels disrupt neuroendocrine regulation, triggering sleep disturbances. Additionally, sedentary behavior may indirectly worsen sleep quality by increasing anxiety and depression [32,33].

The impact of physical activity on inflammatory markers and sleep disorders in osteoarthritis (OA) patients is twofold. Moderate physical activity strengthens periarticular muscles, improves joint stability, promotes blood circulation, and reduces inflammatory markers by activating the body's anti-inflammatory response and releasing neurotransmitters like endorphins, which alleviate pain and improve mood and sleep [34]. However, excessive physical activity can increase joint stress, worsen inflammation, elevate inflammatory markers, and trigger sleep disorders. Additionally, improper physical activity timing, such as vigorous activity near bedtime, can disrupt sleep by keeping the body in an excited state [35].

Finally, we found a non-linear correlation between blood inflammatory marker levels and sleep disorders, with inflammatory markers in the blood being positively correlated with sleep disorders after reaching a threshold. When we excluded individuals receiving anti-inflammatory treatments in the analysis, blood inflammatory markers were not associated with sleep disorders before reaching the threshold, but after reaching the threshold, blood inflammatory markers were positively correlated with sleep disorders. Several biological mechanisms may explain this relationship [36].

Neuroinflammation disrupts the brain's sleep regulation by causing neurotransmitter imbalances, which affect melatonin secretion and the sleep-wake cycle. Inflammation also activates glial cells, releasing excessive mediators that further disrupt neuronal activity and sleep [37,38]. In OA patients, chronic pain and inflammation lead to fluctuations in stress hormones like cortisol, which impair circadian rhythms and sleep structure, contributing to sleep disorders [32]. Additionally, inflammation excites the autonomic nervous system, keeping the body in a heightened state and hindering sleep [39]. In OA patients, sedentary behavior and physical activity influence sleep quality by affecting inflammatory markers. While sedentary behavior may increase inflammatory markers and worsen sleep, moderate physical activity can reduce inflammation and improve sleep quality. Clinically, patients should be guided to manage sedentary time and engage in appropriate physical activity to improve sleep and quality of life. Further research is needed to explore the complex relationships between OA, lifestyle factors, inflammation, and sleep disorders.

This study has several limitations. First, due to its cross-sectional design, causal relationships between sedentary behavior, physical activity, inflammatory markers, and sleep disorders cannot be established. These factors vary over time, making it difficult to determine cause and effect. Longitudinal studies with long-term follow-up of OA patients are needed to explore the mediating role of inflammatory markers. Second, our assessment of sedentary behavior and physical activity relied on self-reported data, which may introduce bias due to varying patient perceptions. More accurate measurements, such as using fitness trackers or accelerometers, are needed. Inaccurate assessments may affect the reliability of the analysis of inflammatory markers as mediators. Finally, the sample primarily consisted of OA patients, so future studies should include larger, more diverse populations to confirm the mediating role of inflammatory markers in sleep disorders, ensuring broader applicability of the findings.

## 5 Conclusion

This study reveals a significant association between sedentary time, physical activity frequency, and sleep disorders in OA patients. Blood inflammatory markers (e.g., NLR, LLOD) correlate with sleep disorder severity. Mediation analysis shows that inflammatory markers partially mediate the link between sedentary behavior and sleep disorders, while fully mediating the effect of physical activity on sleep improvement. The study highlights significant differences in how continuous versus intermittent physical activity regulates the inflammation-sleep axis in OA patients. Future research should examine the dynamic relationship between circadian rhythms and inflammatory fluctuations to develop personalized sleep interventions based on inflammatory biomarkers. Multi-center randomized controlled trials are needed to verify the combined effects of physical activity and anti-inflammatory therapies on sleep disorders, strengthening evidence for comprehensive clinical management strategies.

## Ethics approval and consent to participate

Not applicable.

## Clinical trial number

Not applicable.

## Consent for publication

The publication of this manuscript is approved by all authors.

## Availability of data and materials

Not applicable.

## Authors' contributions

YC Song had full access to all the data in the study and takes responsibility for the integrity of the data and the accuracy of the data analysis. Study concept and design: YC Song, B Luo, Y X Ouyang. Acquisition of data: B Luo, Z Y Huang, R Z Chen, S Zhang, Y T Chen, M D Cao, L Kong. Analysis and interpretation of data: B Luo, Z Y Huang, R Z Chen, X Wu. Drafting of the manuscript: B Luo, Z Y Huang, S Zhang.

Critical revision of the manuscript for important intellectual content: YC Song, B Luo, Z Y Huang, Q S Guo, Y X Ouyang, H Wang. Statistical analysis: B Luo, Z Y Huang, X Wu, Q S Guo, M D Cao. Supervision: YC Song, H Wang. All authors read and approved the final manuscript.

## Funding

This study was supported by the 10.13039/501100003453Natural Science Foundation of Guangdong Province (2020A1414010368), 10.13039/501100020084Guangzhou Municipal Science and Technology Bureau (SL2022A04J00907), and Guangdong Yijia Regenerative Medicine Research Institute Co., Ltd. (HXJF202002).

## Competing interests

The authors declare that they have no competing interests.
